# A Nonparametric Procedure for Defining a New Humoral Immunologic Profile in a Pilot Study on HIV Infected Patients

**DOI:** 10.1371/journal.pone.0058768

**Published:** 2013-03-22

**Authors:** Chiara Brombin, Lorenzo Diomede, Daniela Tudor, Anne Sophie Drillet, Claudia Pastori, Elena Poli, Agostino Riva, Caterina Uberti-Foppa, Massimo Galli, Clelia Di Serio, Morgane Bomsel, Lucia Lopalco

**Affiliations:** 1 Centro Universitario di Statistica per le Scienze Biomediche, University Centre of Statistics in the Biomedical Sciences, Vita-Salute San Raffaele University, Milan, Italy; 2 Immunobiology of HIV Unit, Division of Immunology, Transplantation and Infectious Diseases, San Raffaele Scientific Institute, Milan, Italy; 3 Institut National de la Santé et de la Recherche Médicale, U1016, Institut Cochin, Paris, France; 4 Centre national de la recherche scientifique Joint Research Unit 8104, Paris, France; 5 Entrée Muqueuse du VIH et Immunité Muqueuse Institut Cochin, Université Paris Descartes Cité Paris Sorbonne, Paris, France; 6 University of Milan, Milan, Italy; 7 Department of Infectious Diseases, San Raffaele Scientific Institute, Milan, Italy; Helmholtz Zentrum Muenchen - German Research Center for Environmental Health, Germany

## Abstract

This work aims at identifying a set of humoral immunologic parameters that improve prediction of the activation process in HIV patients. Starting from the well-known impact of humoral immunity in HIV infection, there is still a lack of knowledge in defining the role of the modulation of functional activity and titers of serum antibodies from early stage of infection to the development of AIDS. We propose an integrated approach that combines humoral and clinical parameters in defining the host immunity, implementing algorithms associated with virus control. A number of humoral parameters were simultaneously evaluated in a whole range of serum samples from HIV-positive patients. This issue has been afforded accounting for estimation problems typically related to “feasibility” studies where small sample size in each group and large number of parameters are jointly estimated. We used nonparametric statistical procedures to identify biomarkers in our study which included 42 subjects stratified on five different stages of HIV infection, i.e., Elite Controllers (EC), Long Term Non Progressors (LTNP), HAART, AIDS and Acute Infection (AI). The main goal of the paper is to illustrate a novel profiling method for helping to design a further confirmatory study. A set of seventeen different HIV-specific blood humoral factors were analyzed in all subjects, i.e. IgG and IgA to gp120IIIB, to gp120Bal, to whole gp41, to P1 and T20 gp41 epitopes of the MPER-HR2 region, to QARILAV gp41 epitope of the HR1 region and to CCR5; neutralization activity against five different virus strains and ADCC were also evaluated. Patients were selected on the basis of CD4 cell counts, HIV/RNA and clinical status. The Classification and Regression Trees (CART) approach has been used to uncover specific patterns of humoral parameters in different stages of HIV disease. Virus neutralization of primary virus strains and antibodies to gp41 were required to classify patients, suggesting that clinical profiles strongly rely on functional activity against HIV.

## Introduction

Host humoral immunity is differently involved in fighting HIV infection during progression from first virus contact to overt infection, including evolution from acute to chronic course. Antibodies are key players and take part in different aspects of host-virus interaction, especially those directed at the HIV-1 envelope glycoprotein subunits, gp120 and gp41 that interferes with the initial entry events. However, due to high HIV-1 envelope sequence natural variability, generation of high-titer neutralizing antibodies has been proven difficult. Generically, high-titer of serum neutralizing antibodies have been considered a correlate of HIV protection, although they only appear after months or years of infection, possible upon a deep antigen stimulation sustained by high virus load [1].

Hence, serum antibodies raised against HIV-1 envelope proteins during acute infection are usually ineffective to prevent the establishment of infection, their selective pressure does not control–but can even sustain–autologous virus escape [2].

Subsequent waves of antibodies targeting specific, functional epitopes maintain virus drift through their increased affinity and keen targeting [3]. Antibodies to conserved, neutralizing domains (e.g., the gp120 carbohydrate, MPER) develop heterogeneously in chronic infection, and are not always neutralizing, despite specific of neutralizing motifs. It suggests that generation of neutralizing antibodies is controlled by many factors, such as host genetics, modes of antigen exposure, antibody affinity maturation, and immune tolerance [3]. Other serum humoral responses, bridging innate and adaptive immunity, such as those mediated by binding, non-neutralizing antibodies through Fc receptor, complement cascade and effector killer cells, were also observed in acute infection [3]. Some of these, such as ADCC (Antibody-Dependent Cellular Cytoxicity) and ADCVI (Antibody-Dependent Cell-mediated Virus Inhibition), were found more significant than virus neutralization in protection, being associated with reduced viremia and better virus control. Indeed, sera from HIV controllers showed a significantly higher ADCC activity, highlighting the specific role of this mechanism in long-term HIV control [4], [5].

In this study we aim at providing a multivariate nonparametric analysis to combine information from serum envelope-specific antibodies targeting key HIV epitopes, ADCC and infectivity reduction against a panel of viruses. These parameters are measured in various groups of HIV-positive patients at different stages of infection. Moreover, as anti-CCR5 antibodies have been associated to protection, we checked for such antibodies in all subjects, to establish whether such antibodies could represent a marker of resistance to HIV infection or progression of the disease [6], [7].

The classification and regression tree methodology (CART) developed by Breiman et al. [8] has been applied to use a combined information derived from the whole set if parameters for identifying possible biomarkers. CART is a non-parametric technique for partitioning a population/sample into subgroups. Actually it operates a selection of the explanatory variable, useful to construct the tree, on the basis of their capacity in identifying the most homogeneous subgroups.

This strategy allowed to define profiles of antibody reactivity specific to each group and identify specific “humoral signatures” which may not only have diagnostic relevance, but also identify possible protective parameters by new combinations of humoral factors.

Thus, the paper focuses on the search for a novel biomarker combination that might be assessed as predictive on confirmative larger studies for the evolution of disease in HIV patients. Considering a biomarker panel with respect to the single biomarker improves the diagnosis and may serve as an early warning system of risk for future adverse AIDS outcomes. This goal cannot be achieved by common ROC curve approach, that searches for the greatest separation of two probability curves, leading to the likelihood of distinguishing a sick patient from healthy one based on a unique trait only. Thus ROC method can be used for comparisons only to decide whether one marker allows for better screening between diseased and not diseased subjects with respect to another marker.

Conversely, we constructed a parameters panel on the basis of different parameters combination.

## Results

### Patients and Experimental Design

Forty-two serum samples from HIV-infected patients at different clinical stages (10 AI, 7 AIDS, 8 EC, 7 HAART+, 10 LTNP) were examined (Table 1). Patients in AI and HAART+ subgroups received appropriate therapy. AI subjects were evaluated both before and after 4–8 months of treatment. All humoral parameters studied were evaluated in parallel with CD4+ T cell count and viral RNA load (Table 1). Eighteen humoral parameters concerning specific IgG and IgA profiles, as well as functional antibody assays were performed in all samples, for a total of 23 individual parameters (Table 2), treated as continuous or as categorical variables; twenty-three endpoints actually underwent statistical analyses.

**Table 1:** Characteristic, clinical status of the studied population.

	N°	Risk factor	Mean Age (Range)	Sex	Therapy	Mean HIV RNA (Range)	Mean CD4 (Range)
AI	10	HE, HO	31 (17–49)	6M, 4F	NO	88450 (<37–190000)	621 (204–1069)
HAART	7	HE, HO	42 (32–61)	9M, 1F	YEStn:a	<37	738 (517–2184)
AIDS	7	HO, IVDU	37 (33–46)	5M, 2F	NO	73637 (21079–440000)	226 (141–235)
EC	8	HE, HO, IVDU	51 (34–67)	4M, 4F	NO	<37	1082 (511–1880)
LTNP	10	HE, HO, IVDU, TR	49 (43–71)	7M, 3F	NO	2680 (<37–11000)	821 (513–1515)

In the “Risk factor” column, HE is an abbreviation for “Heterosexual”, HO for “Homosexual” and TR for “Transfusion”, while IVDU stands for IntraVenous Drug Users.

1 2NRTI, 3NRTI, NNRTI+IP,3NRTI+IP, 2NRTI+2IP.

**Table 2:** Parameters used in the study.

Humoral parameters	Viruses used for the neutralization					
Protein Tot.	Region	Isotype	Viruses	Clade	Tropism	Target cells
CCR5	ECL1tn:b	IgG-IgA	SOS 140	Lab Strain B	R5	U87
HR2-gp41	P1-HR2tn:c	IgG-IgA	SF162	Lab Strain B	R5	TZMbl
HR2-gp41	T20-HR2tn:d	IgG-IgA	QH0692	Primary B	R5	TZMbl
HR1-gp41	HR1tn:e	IgG-IgA	PVO	Primary B	R5	TZMbl
gp41	Consensus B	IgG-IgA	AC10	Primary B	R5	TZMbl
gp120	IIIB	IgG-IgA	ZM214	Primary C	R5	TZMbl
gp120	Bal	IgG-IgA				
ADCC		IgG				

1 aa sequence: YAAAQWDFGNTMCQ.

2 aa sequence: QNQQEKNEQELLELDKWASLWNWFNITNWYIK.

3 aa sequence: YTSLIHSLIEESQNQQEKNEQELLELDKWASLWNWF.

4 aa sequence: GIKQLQARILAVERYLKDQQLLG.

### Humoral Parameters

The study focused total IgG and IgA, and HIV-specific IgG and IgA to gp120Bal (R5 strain), gp120IIIB (X4 strain), gp41, three different gp41 epitopes (QARILAV within HR1, T20 and P1 both in the MPER domain within HR2, already described as target of neutralizing antibodies in humans) and finally, anti-self antibodies to CCR5 coreceptor, which were previously found in LTNP Table 2 [6], [7], [9]–[11].

**Figure 1:**
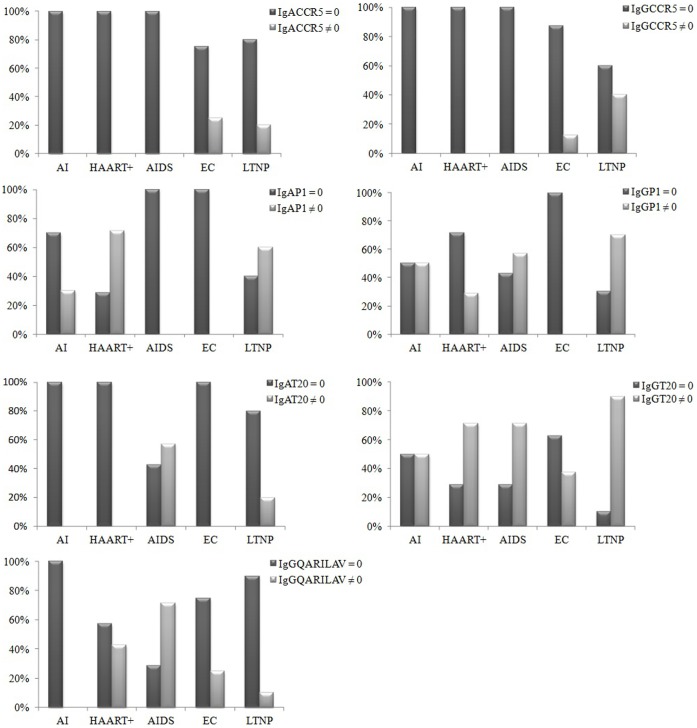
Distribution of binding antibodies in all studied groups. Graphical representation of non-continuous variables, i.e. parameters found in some subjects or groups only. Seven variables, including IgG and IgA to specific gp41 epitopes (P1, T20, QARILAV) and antibodies to CCR5 coreceptor. First bar in each pair shows proportion of values found equal to 0 in the analysis, the second illustrates values different from 0. Chi-square association test between categorical variables only found a significant association between IgGT20 and IgGP1 (p-value = 0.001).

Figure 1, reports the distribution of seven antibodies, namely IgG and IgA to specific gp41 epitopes and to CCR5 coreceptor. These measurments are categorical. Their descriptive analysis revealed that:

- Both IgA-CCR5 and IgG-CCR5 were uniquely observed in some EC and LTNP patients.
- IgG-QARILAV were mainly found in AIDS patients, but not in AI group. No such IgA were isolated in any group.
- IgG-P1 peptide were found in all groups but EC, corresponding IgA in all groups except EC and AIDS.
- IgG-T20 were observed in all groups, such IgA were only in LTNP and in AIDS patients.

**Figure 2:**
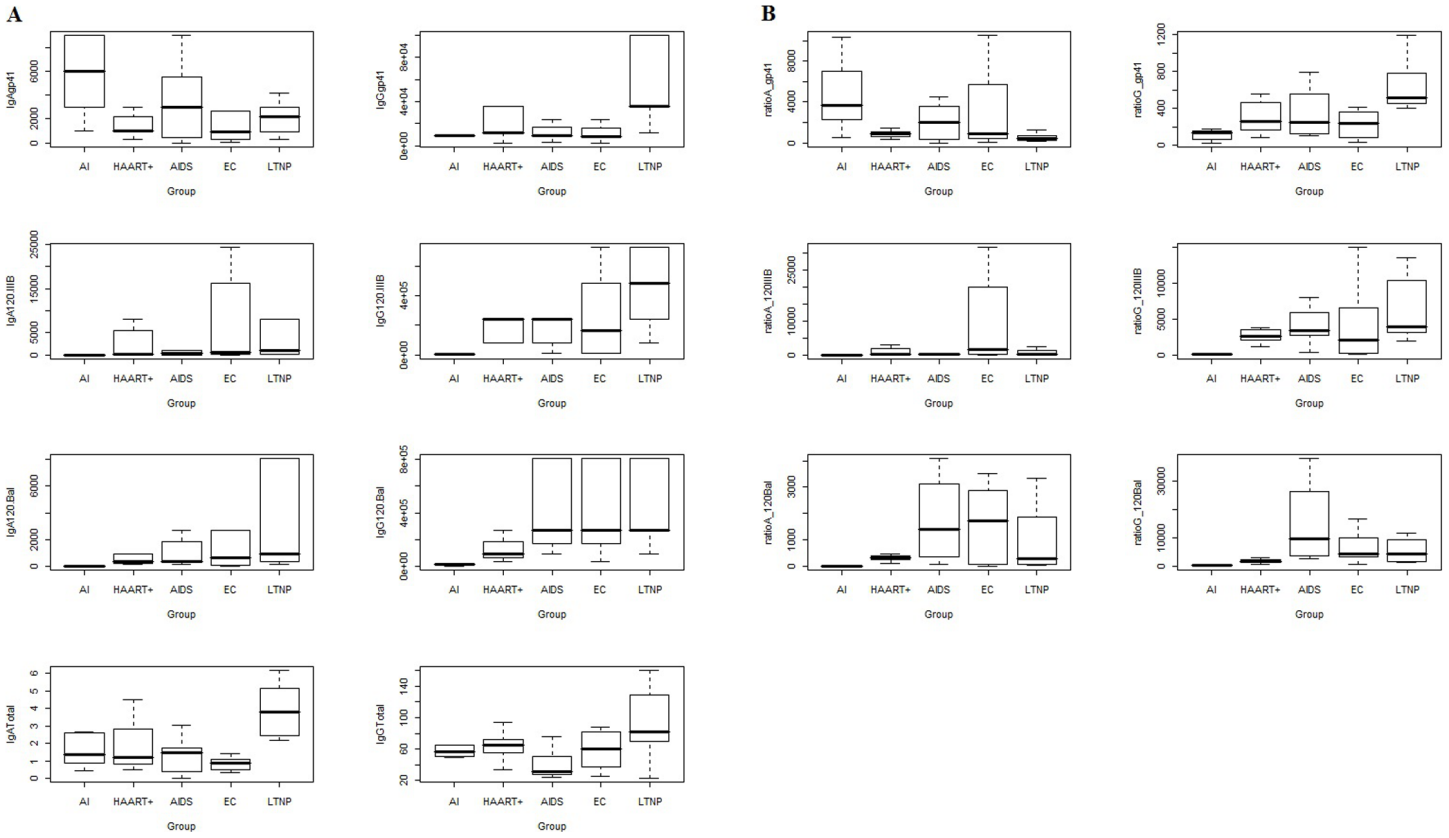
Graphical representations of humoral parameters. Panel A shows antibody concentrations, Panel B reports ratio of antibodies to total IgG or IgA.

Continuous measurements were provided on total immunoglobulins isotypes and specific IgA and IgG to whole gp120 and gp41 (see graphical representation in Figure 2, panel A).

The corresponding “ratio variables” were obtained by normalizing raw values of continuous variables over the total amount of the immune response (see also Statistics section).

With respect to the distributions of ratio variables (Figure 2, panel B), Gp41 showed higher variability in IgA than IgG (except for LTNP patients). Distributions of ratio variables, both IgA-gp120IIIB and IgG-gp120IIIB, when scaled by the respective total response, had similar behavior both in terms of location and dispersion, especially in AI patients. IgA-gp120IIIB showed higher dispersion that IgG-gp120IIIB in HAART patients. IgA-gp120Bal and IgG-gp120Bal showed a similar behavior in all patents’ groups (Figure 2, panel B).

### Antibody Mediated Functional Activities

**Figure 3:**
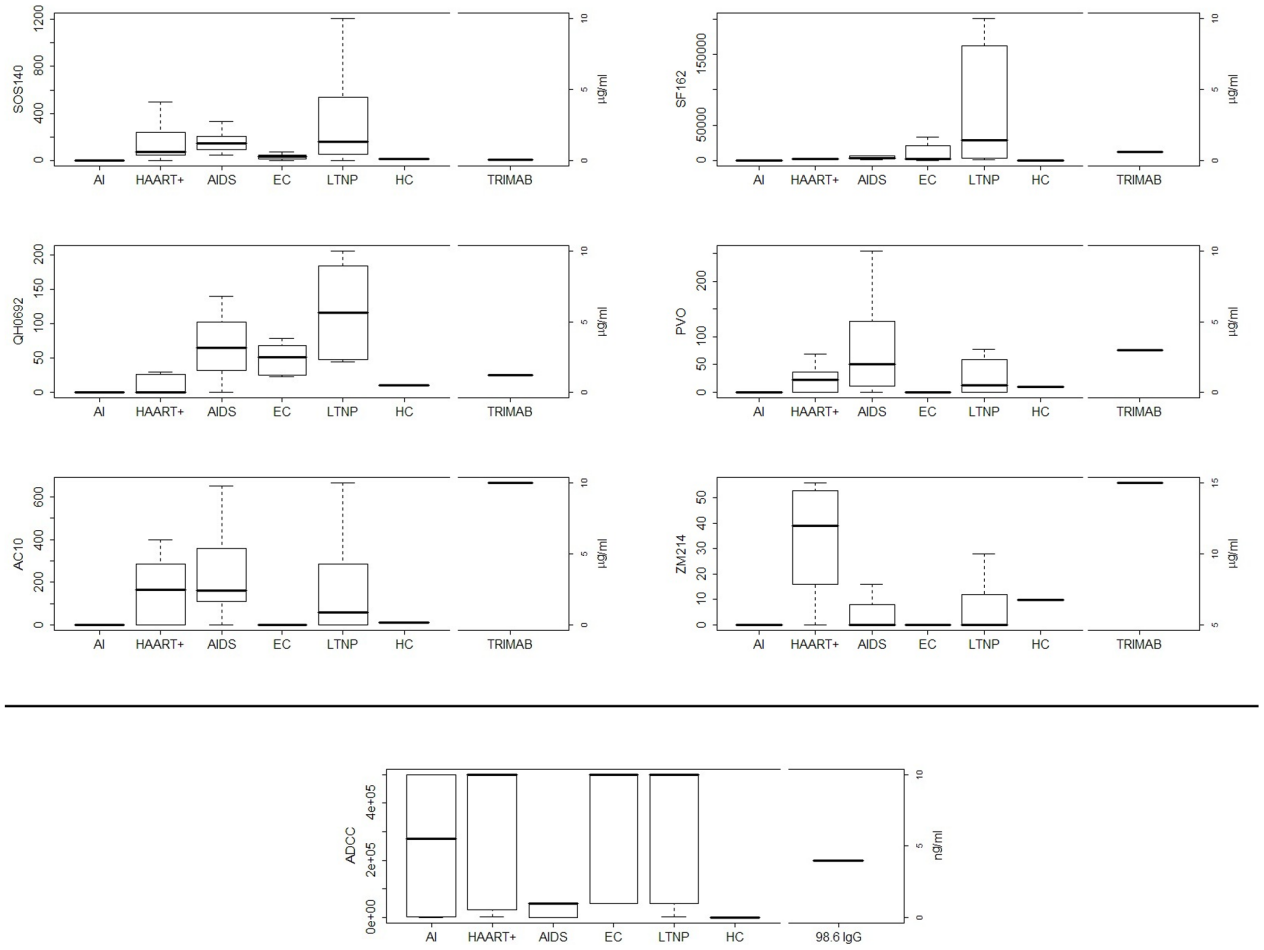
Antibodies functional activities including neutralization and ADCC in the study population. Neutralization profiles obtained with six different viruses in two assays which use U87 or TZM.bl cell lines as target cells are shown. SOS140 was used to infect U87 cell line, the other viruses including SF162 (lab strain), three clade B (QH0692, PVO and AC10) and a clade C (ZM214) viruses were used to infect TZM.bl. HC (healthy controls) pooled sera were used as negative control and TRIMAB monoclonal antibody mix was used as positive control. The values are expressed as IC50 (serum dilution 1/n for all samples or µg/mL for TRIMAB leading 50% of infectivity reduction). The last panel, in the middle, shows ADCC activity by all five groups of HIV seropositive subjects. HC (healthy controls) pooled sera and 89.6 IgG were used as negative and positive controls, respectively. The values are expressed as titers.

Neutralizing potential of all sera was assayed on six virus strains; two methods with two different cell lines as target cells, TZM-bl and U87, were used, to enhance assay sensitivity. Distributions of viruses are shown in Figure 3.

SOS140 was used to infect U87 cell line, the other viruses including SF162 (lab strain), three clade B (QH0692, PVO and AC10) and a clade C (ZM214) primary isolates, were used to infect TZM.bl cells. HC (healthy controls) pooled sera were used as negative control and TRIMAB monoclonal antibody was used as positive control.

Neutralization activity was poor in AI and in EC groups and remarkable in LTNP and AIDS patients in the four primary viruses (QH0692, PVO, AC10 and ZM214), suggesting it could be induced and enhanced by long-lasting exposure to viral antigens (Figure 3).

Conversely, ADCC activity was observed in patients exposed to very low levels of circulating viral antigens, such as AI, EC, LTNP and in the group receiving HAART, while it was very low in AIDS patients; hence, a low amount of viral load could be crucial to induce and maintain this type of humoral response over time: strikingly, AI and EC groups showed high ADCC vs poor neutralizing activity (Figure 3).

### Classification Trees

**Figure 4:**
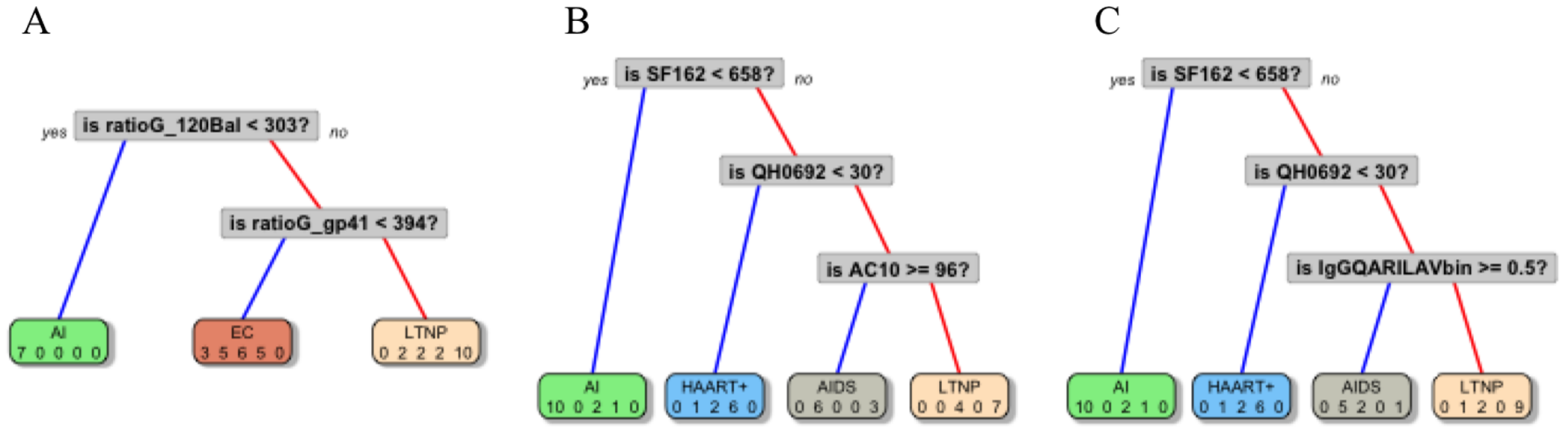
Classification trees resulting from CART analysis, performed with binding antibodies (A), functional activities data data (B) or antibody mediated binding and functional activities (C). Numbers inside grey squares indicate the discrimination level and are expressed as dilution 1/n.

The joint effect of endpoints in classifying patients into clinically-important groups was assessed through Classification and Regression Tree analysis (CART) [8]. To this purpose, all the humoral parameters, the virological endpoints, and patients groups were included in the analysis. Figure 4 shows resulting CARTs and decision rules, displayed in a simplified version; numerical values correspond to antibody dilutions or to neutralizing titer, respectively.

#### CART on binding antibodies (before pruning)

Two out of 13 variables entered in the model were selected to discriminate/classify patients' groups. Only information from IgG-types antibodies were required to classify patients (Figure 4, A). The ratio-IgG-gp120Bal (when ratio-IgG-gp120Bal<303) allowed to correctly classify 70% AI patients; using the ratio-IgG-gp41 (when ratio-IgG-gp41≥394), 100% LTNP were correctly classified. When ratio-IgG-gp41<394, 75% EC, 71.4% AIDS and 71.4% HAART+ patients were classified, respectively. We concluded that AIDS, HAART+ and EC patients shared a similar pattern, completely different from LTNP patients.

#### CART on functional activities (before pruning)

Three out of 7 variables entered in the model were selected to discriminate/classify patients' groups. SF162 neutralization classified all AI patients (Figure 4, B); information on QH0692 identified 85.7% HAART+ patients. AC10 data discriminated 85.7% AIDS patients (when AC10≥96) and 70% LTNP patients (when AC10<96).

#### CART on antibody mediated binding and functional activities (before pruning)

Three out of 20 variables entered in the model were selected to discriminate/classify patients’ groups. Notably, two out of three branches of this latter tree resulted super-imposable to the previous CART tree, where only virus data were used (Figure 4, C). SF162 data classified AI patients; information on QH0692 also identified 85.7% HAART+ patients (when QH0692<30). The binary variable IgG-QARILAV, distinguished 71.4% AIDS patients (when IgG-QARILAV≠0) and 90% LTNP patients (when IgG-QARILAV = 0).

CART trees were implemented using R software [12] (package rpart, see http://www.R-project.org). Default “cost complexity” factor cp = 0.01 was chosen.

**Figure 5:**
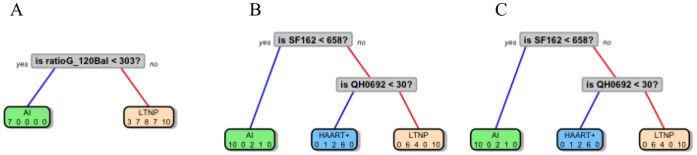
A pruned version of the optimal trees.

To determine whether trees were appropriate or if some of the branches needed to be subjected to pruning, thus avoiding overfitting and controlling the size of the decision trees, we have examined the cross-validated error results (“xerror”, see Table **S1** in File S1), and we have selected the “complexity parameter” (cp parameter, see Figure **S1 in File S1**) associated with the smallest cross-validated error; then we placed it into the function “prune” to prune the tree. Resulting trees after pruning are shown in Figure 5 and described below.

#### CART on binding antibodies (after pruning)

One out of 13 variables entered in the model were selected to discriminate/classify patients' groups. Only information from IgG-gp120Bal antibody was maintained after pruning. Hence when ratio-IgG-gp120Bal<303 70% AI patients were correctly classified, when ratio-IgG-gp120Bal≥303, 100% LTNP were correctly classified (Figure 5, A).

#### CART on functional activities (after pruning)

Two out of 7 variables entered in the model were selected to discriminate/classify patients' groups (Figure 5, B). SF162 neutralization classified all AI patients. QH0692 data discriminated 85.7% HAART+ patients (when QH0692<30) and 100% LTNP patients (when QH0692≥30).

#### CART on antibodies mediated binding and functional activities (after pruning)

Two out of the 20 variables entered in the model were selected to discriminate/classify patients' groups (Figure 5, C). SF162 data classified AI patients; information on QH0692 also identified 85.7% HAART+ patients (when QH0692<30) and 100% LTNP patients (when QH0692≥30). After pruning, the last two trees were found to be equal, because only information on virus was selected to classify patients.

In general, the pruning procedure is applied to avoid overfitting the data. However, in our data set, only few variables were selected in the “growing phase” of the tree. Then during the “pruning phase” only the last node was deleted and, as a result, there was an increase the misclassification error. Hence, in our particular case, pruning phase was not really effective and tree obtained before pruning are more informative.

Finally, we faced the validation issue. Since small sample size represents a difficult feature to afford a cross-validation procedure, we evaluated the performance of the tree in terms of misclassification rate by bootstrapping 1000 trees based on the same covariates of our analyses (ratio of antibodies, viruses and combination of both). This procedure led to classifying correctly 70% of the observation for each tree, that can be considered a robust proportion once related to the initial sample composition.

## Discussion

The definition of key protective factors conferring optimal in vivo protection is still debated. At our knowledge, this is the first study where a number of humoral parameters were simultaneously evaluated in a whole range of serum samples from HIV-positive patients. Despite the small sample size, due to the clear definition of the research objective, this study can be considered as “pilot”, and design new diagnostic strategies for larger studies. We assessed antibodies to specific HIV epitopes, to CCR5, virus neutralization and ADCC, and specific statistical tools were applied, aimed at defining specific ``humoral signatures biomarkers characteristics of each clinical status. Notably, all parameters were simultaneously tested, using the same methods and the same source of reagents to minimize variability, which is a relevant issue in HIV research, when immune correlates of protection have to be uniquely identified.

Since the analysis was aimed at investigating variables combinations in defining diagnostic categories, a multivariate approach was chosen. While standard ANOVA approach examines covariates effect one by one, the use of CART procedure allowed for analyzing multiple humoral parameters, classified as categorical or continuous, for the identification of antibodies patterns. Correlation of particular immune response profiles and clinical stages of HIV infection suggests novel immunopathway(s) that could be exploited to improve immune control of HIV.

By means of Trees representation, it can be easily visualized that IgA and IgG antibodies exhibited different, sometimes opposite, patterns throughout patients groups. AI, AIDS and EC groups showed similar patterns on IgA total response; IgG total response showed high variability, especially in AI, EC and LTNP group. Antibodies to CCR5 were only observed in some patients controlling the infection (EC and LTNP), suggesting that they could be elicited late in the course of infection and that a low-dose antigen exposure could allow their maturation. Interestingly, anti-MPER antibodies showed different patterns in LTNP and EC groups, suggesting that their generation might depend on the presence of circulating virus. Moreover, IgG-T20 and IgG-P1 were different in all groups, suggesting that humoral responses differently addressed 2F5 and other MPER epitopes, variously assorted within partly overlapping T20 and P1 sequences (see Tables 1–2). In fact, P1 (aa 650–684) is a 35 amino acid long peptide, which adopts a 3D conformation that could improve recognition by antibodies binding conformational epitopes; both lipid environment and pH are critical for determining physiological solution structure of P1 epitopes without altering the native 3D-structure of MPER [13]. P1 contains a bent in its C-terminal region, just placed at the level of the 4E10 epitope; furthermore, 2F5 and 4E10 IgG recognize P1 better than their nominal epitopes ELDKWA [14] and NWFDIT (unpublished results). In contrast, T20 (aa 638–673) has a shorter C-terminus compared to P1 (Tables 1–2), it therefore does not contain the hydrophobic region that participates in the structure of the W-rich region present in P1. In addition, T20 does not contain the 4E10 epitope and would lack proper 3D structure [15], [16].

Therefore it would be poorly recognized by antibodies against conformational epitopes, i.e., the opposite condition observed with P1 peptide. IgG-QARILAV antibodies appeared in later stages of infection, were absent in AI and had the highest titers in the AIDS group. This fact may imply very-low dose antigens, or defective virions could have triggered such antibody production. Interestingly, virus particles generated in the late phase of the infection, including AIDS, could differ from those sustaining early infection; this finding was usually observed in isolates undergoing many subsequent infection cycles in vitro, and therefore might explain the lack of such antibodies in AI [17]. A key point emerging from CART analysis is that data about specific IgG-gp41 and viruses were both required to define patients’ status (Figure 4, C). The protective role of anti-gp120 antibodies, taken as a whole, agrees with previous studies, where gp120-binding antibodies were associated to virus control via ADCC and ADCVI, with different antibody subsets mediating virus neutralization in terms of specificity and of timing of generation [5], [18], [19]. Notably, these studies did not take into account specific binding properties and timing of generation of antibody subsets to gp120 and gp41, neither IgA contribution was evaluated. Antigen exposure was required to sustain neutralization activity, as it was found limited or absent in AI and in EC groups but high in LTNP, HAART and AIDS. SF162 (lab strain B-R5) was the most sensitive strain in neutralization assays; neutralizing titers to other clade B strains were by far lower (e.g. titers <200 vs PVO), suggesting that each virus within the panel had a different sensitivity to neutralization even within the same clade. This is also true for clade C ZM214 strain, which was neutralized at very low titers (titers <50), probably due to the fact that each patients' group can display a different neutralizing potential towards a given virus strain. This might depend on the exposure to clade-specific strains, but also on modes and duration of antigen exposure. In fact, in some studies, LTNP showed very high neutralizing titers to different virus strains, while EC patients only achieved poor or no neutralizing activity at all [4], [20], [21]. Differently from neutralizing antibodies, ADCC was observed since early stages of infection while declining in AIDS, suggesting it plays a role in controlling HIV in AI and in later stages of infection. The tree based on virus neutralization data achieved a better group classification (Figure 4, B); strikingly, it was partly identical to that generated with both virus and antibody data, thus confirming that functional ability to neutralize viruses correlated more accurately with clinical classification of patients than the presence of specific antibody subsets, or with antibody proportion over total immunoglobulin content. Although we relied on this statistical technique due to the numerous advantages listed above and in the Statistics Section, however we are aware that CART methods suffer a big limitation since they split only by one variable. This may imply that if the data set has more complex structure then CART may not catch it correctly. Indeed, key points emerging from CART analysis were the following:

- gp41 plays a crucial role in determining a stage-specific signature, as IgG to whole gp41 or to its QARILAV epitope appeared in both “binding antibodies only” and in “combined” trees (Figure 4, A and C);
- virus neutralization, i.e. functional activity, was more predictive than antibody binding in defining clinical profiling; after pruning the tree, neutralization of two virus strain (a lab and a primary strain) were retained in the model in “functional activities only” and especially in “combined” tree, while the other humoral parameters were fully excluded (compare Figure 4, B and C, vs Figure 5, B and C);
- ADCC, albeit observed in some clinical groups, was less significant than neutralization in terms of patients classification;
- antibodies to gp41 and neutralizing activity could offer a reliable tool in clinical stratification of small-medium sized panels of patients;
- CART classification of parameters did not select immune parameters according to their potential in terms of immune protection, but only by a clinical point of view.

Thus, we recommend this methodology as suitable to provide diagnostics guidelines in pilot studies.

Small studies with all the trappings of a major study, such as randomization and hypothesis testing may be labeled a “pilot” because they do not have the power to test clinically meaningful hypotheses. There are two major parametric assumptions, which are routinely violated in pilot studies: sample size, and normal distribution of the dependent variable. Although nonparametric techniques do not require the stringent assumptions associated with their parametric counterparts, this does not imply that they are assumption free. CART analysis may be seen as an automatic “machine learning” method that produces a decision tree that can be used for explorative purposes in studies with small sample size within each cluster and allows to group subjects into more homogeneous groups, using combinations of variables.

## Materials and Methods

### Sample Description

Ten Acute Infections (AI), 7 HAART treated patients (HAART+), 7 AIDS patients at terminal stage of the diseases (AIDS) naive for antiretroviral drugs, 10 Long Term Non Progressors (LTNP), 8 Elite Controllers (EC), as shown in Table 1. The inclusion criteria for LTNP were: 1. certified HIV-1 seroconversion at least 7 years before enrollment; 2. asymptomatic HIV-1 infection and good health conditions; 3. peripheral CD4+ T cell counts always >500 cells/mm^3^; 4. never receiving antiretroviral therapy [22]. EC were defined as HIV-1 infected patients able to exert spontaneous control of viremia for at least 2 consecutive years in the absence of HAART and viral load persistently <37 copies/mL [23]. HAART+ patients had CD4 counts >500 cells/mm^3^ on antiretroviral treatment for at least 24 and not more than 30 months with chronic and progressive infection, but without previous AIDS defining disease. The similar length of time of suppressive therapy in HAART treated patients has been chosen in order to minimize possible differences in immune status. AIDS patients exhibited one or more AIDS defining diseases and CD4+ T cell counts <250 cells/mm^3^ (these patients were recruited between 1985–1993, before HAART era, and they died after enrollment). In regard to AI, the eligible patients had to fulfill at least one clinical criterion (signs and symptoms of acute retroviral syndrome-ARS; signs and/or symptoms of ARS during the previous 60 days; exposure to HIV in the previous three months and a negative test in the previous six months) and one laboratory criterion (detectable plasma HIV-RNA; only gp120, gp160±p24 bands at Western blotting; a low positive ELISA with increasing reactivity over time). The AI population was analyzed at the onset of disease and six months after receiving antiretroviral therapy. No statistical differences were found for sex and age among the different populations. Table 1 summarizes the clinical status of the study populations.

Pooled sera from 10 Healthy-Controls (HC) not exposed to HIV were used as negative controls in all assays.

All studied populations were recruited at the Department of Infectious Diseases of the San Raffaele Scientific Institute or at the Infectious Disease Clinic of the University of Milan at L. Sacco Hospital.

The institutional review board and the local ethic committee of San Raffaele Scientific Institute named “Comitato Etico della Fondazione Centro San Raffaele del Monte Tabor-Istituto Scientifico Ospedale San Raffaele” and of University of Milan named “Comitato Etico Locale per la Sperimentazione Clinica dell'Azienda Ospedaliera Luigi Sacco di Milano” approved the investigations and all subjects gave written informed consent for the study.

### Quantification of Immunoglobulins

Total IgA and IgG in all serum samples were measured with ELISA. Briefly, ELISA plates were coated with a 1∶2000 dilution of a goat anti-human IgA or IgG (100 ul/well) in coating buffer and incubated for 1 h at 37°C. After washing, blocking buffer (1% Skim Milk in Phosphate Buffer, Sigma) was added and plates were incubated for 1 h at 37°C. Serial dilutions of samples and IgA or IgG reference standards (Sigma) were incubated for 1 h at 37°C. After washing, Goat anti-human IgA-Biotin (diluted 1∶5000) or Goat anti-human IgG-Biotin conjugate diluted 1∶2000 (KPL) was added and incubated 1 h at room temperature. Then, Streptavidin-HRP conjugate (Vector Laboratory) diluted 1∶3000 was incubated for 1 h at room temperature. TMB substrate (KPL) was incubated for 5 minutes at room temperature in the dark. Then 10% H_2_SO_4_ was added and plates were read out with a spectrophotometer at 450 nm. Total IgA or IgG concentrations were determined by interpolation, using the calibration line of IgA or IgG reference standards, respectively.

### Binding of Immunoglobulins to Recombinant env Proteins and Peptides

The immunoglobulin fractions were tested in sandwich ELISA to identify binding antibodies to env proteins, as previously described [24]–[26]. The recombinant proteins gp120Bal and gp120IIIB (obtained through the NIBSC, Programme EVA Centre for AIDS Reagents, UK) and the gp41-specific peptides, shown in Table 2 were used. Microwell plates were coated with recombinant proteins (gp120 Bal and gp120 IIIB obtained through the NIBSC, Programme EVA Centre for AIDS Reagents, UK) and gp41-specific peptides (Table 2) at 1 µg/mL by means of overnight incubation in NaHCO_3_/Na_2_CO_3_ buffer. The plates were saturated for 1 h with PBS and 3% bovine serum albumin. The eluted Igs were added and incubated for 1 h at 37°C. Ig binding was demonstrated by means of HRP-conjugated rabbit anti-human IgG and IgA (Dako, Santa Barbara, California, USA). The enzymatic reaction was developed and read at 492 nm. The endpoint titers were defined as twice the optical density (O.D.) obtained in 20 seronegative control subjects.

### Virus Neutralization Assays

HIV-1 pseudovirus stocks were generated by co-transfection of 293-T cells with Env-expressing pCAGGS-based plasmids and a backbone plasmid lacking Env (pNL4-3.Luc.R-E-) and titrated for infectivity in TZM-bl and U87.CD4.CCR5 [9], [10]. Co-transfection generates HIV-1 pseudoviruses that are able to infect cells with a single round infection because the plasmids encode an incomplete HIV-1 genome. Two genetically engineered cell lines, TZM-bl and U87.CD4.CCR5 (NIH AIDS Reagent Program, Germantown, MD) and six HIV-1 strains were used to assess the in vitro neutralizing activity of all the samples. CCR5- and CD4-transfected TZM.bl cell line, genetically engineered to express a Tat-responsive luciferase reporter gene (JC53-bl, NISBC) was used as target cell for HIV-1 neutralization assay, as previously described [27], [28]. Neutralizing activity of heat inactivated sera was evaluated using a panel of five pseudoviruses (NIBSC, Programme EVA Centre for AIDS Reagents, UK), including three Clade B (QH0692, AC10 and PVO), one Clade C (ZM214) and one lab strain (SF162). All sera were tested in another neutralization assay, as previously reported [7], [9], [29]. Briefly, U87.CD4.CCR5 cells (a genetically engineered cell line expressing CD4 and CCR5 as well as the luciferase gene) and an HIV-1 envelope mutant that introduces a disulfide bridge between the gp120 surface proteins and gp41 transmembrane protein (named SOS-gp140) were used. The neutralizing activity is readout by the reduction of luciferase gene expression after a single round of virus infection. Luciferase gene expression is quantified by luminescence and is directly proportional to the amount of virus infection.

### Pseudovirus Production for TZM-bl System

Neutralizing activity of heat inactivated sera from immunized animals was evaluated using a panel of five pseudoviruses (obtained through the NIBSC, Programme EVA Centre for AIDS Reagents, UK) including three Clade B (QH0692, AC10 and PVO), one Clade C (ZM214) and one lab strain (SF162), in a standardized and validated single round infection assay. Stocks of single-round infection HIV-1 Env pseudoviruses were produced by cotransfecting 293T/17 cells with 2 µg of an HIV-1 rev/env expression plasmid and 12 µg of an env-deficient HIV-1 backbone plasmid (pSG3DEnv) using Lipofectamine transfection reagent (Invitrogen). Pseudovirus-containing supernatant was harvested 24 h following transfection, clarified by centrifugation and filtered through 0.45 µm filters, and single-use 1 mL aliquots were stored at −80°C. The 50% tissue culture infectious dose (TCID50) for each pseudovirus preparation was determined by infection of TZM.bl cells as previously described [27].

### Pseudovirus Production for U87.CD4.CCR5 System

A human immunodeficiency virus type 1 (HIV-1) envelope mutant that introduces a disulfide bridge between the gp120 surface proteins and gp41 transmembrane protein (named SOS-gp140) were used. Briefly, plasmid (pCAGGS) was used to express membrane bound envelope (SOS-gp140) of the primary R5 isolate Jr-FL. Vesicular stomatitis virus G (VSV-G) was used as a negative control virus. Pseudoviruses were produced by transfection of 293T cells with pNL4-3-LUC.R-E- and Env expressing pCAGGS-based plasmids. Single round infections were performed using U87.CD4.CCR5, and luciferase activity was measured in the culture supernatants. The SOS virus was incubated with inhibitor for 1 h before being transferred to U87.CD4.CCR5 cells for a further 2 h of incubation. Unbound virus was removed by changing medium, and the culture was incubated for a further 1 h. Cells were plated in a 96-well plate (3×10^4^ cells/well). After 1 h incubation, the growth medium was replaced by fresh growth medium again and the plate was incubated at 37°C for 3 days. Then the plate was washed with PBS and Bright-Glo substrate was added. The cells were allowed to lyse for 2 minutes, and then the supernatant was transferred to a 96-well white plate and readout in a luminometer to quantify the luciferase activity. TCID50 values were calculated according to the method of Reed and Muench [29].

### TZM.bl Neutralization Assay

CCR5- and CD4-transfected TZM.bl cell line (JC53-bl obtained through the NIH AIDS Research and Reference Reagent Program, USA) was used as target cell for HIV-1 neutralization assay, as previously described [27], [28]. Briefly, 3-fold serial dilutions of serum samples (starting from 1∶10 dilution), were plated in duplicate (96-well flat bottom plate) in 10% D-MEM growth medium (100 µL/well). 200 TC ID50 of each pseudovirus were added to each well in a volume of 50 µL and incubated for 1 h at 37°C. TZM.bl cells were then added (1×10^4^ cells/well in a 100 µL volume) in 10% D-MEM growth medium containing DEAE-dextran (Sigma Aldrich) at a final concentration of 11 µg/mL. Assay controls included replicate wells of TZM.bl cells alone (cell control) and TZM.bl cells with virus (virus control). Following a 48 h incubation at 37°C, 150 mL of culture medium were removed from each well and replaced with 100 µL of Bright-Glo luciferase reagent (Promega). After 2 minutes incubation, 150 µL of the cell lysate was transferred to a 96-well black solid plate and luminescence was measured using a Victor Light 2030 luminometer (Perkin Elmer). The 50% inhibitory dose (IC50) was calculated as the serum dilution that induced a 50% reduction in relative luminescence units (RLU) compared to the virus control wells, after subtraction of cell control RLU. A pool of 2F5, b12 and 2G12 neutralizing monoclonal antibodies (TRIMAB) was used at 7.4, 2.5, 0.82 and 0.27 µg/mL, as positive control. A pool of 10 non HIV-related human sera was used as negative control. Results are shown as % of infectivity reduction per each serum after subtracting values observed with pool of pre-immune sera.

### U87.CD4.CCR5 Neutralization Assay

All samples were also tested in another neutralization assay, as previously reported [7], [9], [29]. Briefly, U87.CD4.CCR5 cells were coated in 96-well plates and incubated at 37°C overnight. Serial dilutions of test samples and positive control (MAb 2F5) were prepared in 96-wells plate in triplicate. Three wells for respectively background control and virus control were reserved. The pseudovirus stock JFRL-140WT was diluted in growth medium to a concentration giving 100,000–200,000 CPS, corresponding with 10–30 TCID50/well, and was added to the diluted sample containing wells. After incubation for 1 h, virus-sample mixtures were added to the cell coated plate and incubated at 37°C for 3 days. After washing the cells with PBS, Bright-Glo substrate was added. The cells were allowed to lyse for 2 minutes, and then the supernatant was transferred to a 96-well white plate and readout in a luminometer to quantify the luciferase activity. The percentage of HIV-1 neutralization was calculated as the ratio of CPS of the diluted test samples and CPS of the virus control wells and multiplying by 100 and subtracting the result from 100. Neutralization activities are expressed as the sample concentration or dilution required to reduce the CPS by 50% (IC50).

### Antibody Dependent Cellular Cytotoxicity Assay (ADCC Activity)

ADCC tests were performed as described [30], using CEM-NKr cells coated with each gp41 antiserum, as target cells. NKr-CEM expressing CCR5 and THP1, were obtained from the NIH, AIDS Research and Reference Reagent Program, Germantown, USA. ADCC was performed using a rapid fluorescent-based assay, as described in Guyre *et al.* [31].

Briefly, target cells at 3×10^6^ cells/mL were dually stained with the cytosolic dye CFSE: 5-(and -6-) carboxyfluorescein diacetate succinimidyl ester (Molecular Probes, Eugene, Oregon) at 1mM and with the membrane dye PKH-26 (Sigma, St. Louis, MO) at 1mM for 5 minutes at 37°C. After dual staining, target cells were incubated with the antibodies (serum samples at the indicated dilution, or 2F5IgG as positive control) for 30 minutes at room temperature. Effector cells, THP1, were then added at an Effector:Target (E:T) ratio of 10∶1. When indicated, cells were incubated with irrelevant IgG for 15 minutes prior to their addition in the ADCC reaction. Then, cell co-cultures were centrifuged for 1 minute at 1000 rpm and incubated for 4 h at 37°C. When infected cells were used as targets, they were fixed with 4% paraformaldehyde. Fluorescence profiles of the cell co-cultures were immediately acquired using a Becton Dickinson FACSCalibur. Data analysis was performed using Cytomics RXP software. Flow cytometry dot plot of dual-stained target cells incubated in the same conditions as the effector-target co-cultures, was used to set the gate of living double positive target cells, where the cell membrane was still intact. ADCC was calculated as follows: (% of PKH-26*^high^* CFSE negative cells)/(% of PKH-26*^high^* CFSE negative cells)+(% of PKH-26*^high^* CFSE high cells)×100. When HIV-infected cells were used as target, the ADCC was corrected by the actual amount of HIV-1 infected cells present at the beginning of the assay, as determined by intra-cellular p24 labeling (as indicated above). Percent of ADCC lysis was estimated as the difference in amount killing in presence and absence of a given antibody. Positive control was gp41-specific IgG 98.6 resulting in >22% (SD: +/−3) and specific cell lysis at 100 ng/mL. Titer was defined as the lowest serum dilution or IgG concentration inducing a specific cell lysis >10% [30].

### Statistics

In the preprocessing data phase, final endpoints were considered as continuous, whenever quantitative measurements were taken on a continuous scale, or as categorical endpoints whenever the variables were collapsing on non-continuous values (such as presenting lots of zeros and few measurements different from zero). In this case, dummy variables (0–1 variables) were defined as equal to 1 if the variable takes values different from 0 and 0 if the variable was exactly equal to 0. The different nature of the variables required different statistical tools. Moreover, we used in the analysis some “ratio responses” obtained by normalizing raw values of continuous variables over the total of the immune response (named “Ratio_name of the antibody”).

In general, a nonparametric approach was chosen to allow for small sample size. Figure 2 provides a graphical representation of non-continuous variables, i.e. parameters found in some subjects or groups only. In Figure 2 graphical representations of antibody concentrations and ratios of antibodies to total IgG or IgA.

Classification and Regression Trees (CART) analysis was performed to develop a clinical decision rule to classify HIV patients on the basis of all the measurements, i.e. all the antibodies, clinical endpoints and viruses. Percentage of correctly classified patients provides a measure of accuracy of the derived rule.

CART analysis is a nonparametric and robust data-mining tool that automatically searches for meaningful relationships among variables, thus allowing to discover hidden patterns in complex data and generating reliable predictive models [32].

A clear advantage of general nonparametric procedures over commonly used parametric procedures is that underlying dependence structures among biological variables can be modeled with no need of stringent unrealistic assumptions.

Then, when included in a statistical model, interaction effects involving more than two covariates can be difficult to interpret.

Moreover, in presence of categorical variables taking a large number of values with few observations for each category, convergence problems may occur. Finally, results provided by traditional methods, such as standard multinomial logistic regression, are commonly given in terms of probability and are difficult to communicate to clinicians.

On the other hand, CART trees are data-driven methods easy to interpret, even for non statisticians, and are characterized by low computational complexity. Whenever available, a priori information may be included in CART and may be integrated with other pattern recognition algorithms (e.g., hidden Markov models) [33].

CART analysis allows to construct decision trees useful in classifying subjects into homogeneous groups on the basis of the choice of optimal cut-points of binary, ordinal, or continuous covariates, which maximizes a specific split criterion.

Due to its flexibility in handling complex multivariate/time dependent data, this analysis is gaining popularity in clinical research [34]. Some clear advantages of CART technique are: i) no need of pre-selecting variables in advance. ii) CART algorithm can identify the most significant variables and eliminate non-significant ones. It is possible to test this property by including insignificant (random) variable and compare newly generated tree with the original one, built on initial dataset. iii) results are preserved under monotone transformation of independent variables.iv) CART can easily handle outliers. Outliers in feasibility studies represents a major hurdle. They can negatively affect the results of many standard classification models, such as Principal Component Analysis (PCA) and linear regression. However, these problems can be overcome by CART analysis since, since its algorithm isolates the outliers in a separate node. Actually, CART can be applied for the identification of prognostic factors in many classification problems.

### Decision Trees: General Framework

**Figure 6:**
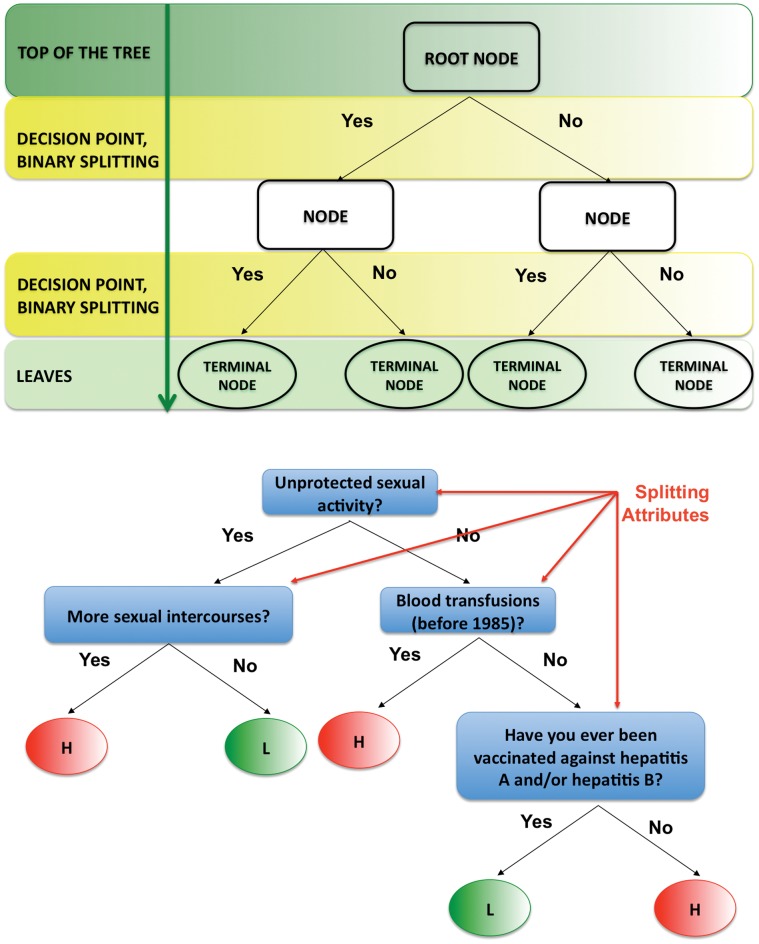
CART toy example. Classification And Regression Trees (CART) are binary decision trees, attempting to classify a pattern by selecting from a large number of variables the most important ones in determining the outcome variable. A decision tree consists of nodes and leaves, with each leaf denoting a class. In a binary tree, by convention if the answer to a question is “yes”, then the left branch is selected and the same question may appear in more than one places in the tree. For example, attributes such as “Unprotected sexual activity”, “More sexual intercourses”, “Blood transfusions (before 1985)” and “Being vaccinated against hepatitis A and/or hepatitis B” can be used to classify people as “L”, low risk of getting infected with HIV and “H”, high risk of getting infected with HIV. Classes (low and high risk of getting infected with HIV) are the outputs of the tree. Attributes (unprotected sexual activity, more sexual intercourses, possible blood transfusions (before 1985) and vaccine against hepatitis A and/or B) are a set of features that describe the data. The input data consists of values of the different attributes. Using these attribute values, the decision tree generates a class as the output for each input data. The top of the tree, or first node, is called the root node, intermediate nodes are the descendant or “hidden” layers and the last level of nodes are the leaf nodes, that contain the final classification.

Now we briefly introduce main ideas behind decision trees and CART algorithms (displayed in Figure 6, A).

A decision tree is made up of nodes and leaves, with each leaf denoting a class/group. Classes, commonly identified with groups of patients relevant from a clinically point of view, are the outputs of a tree.

Clinical endpoints collected throughout the study are the input data.

Each branch of the tree ends in a terminal node and each observation falls into exactly one terminal node. Each terminal node is uniquely defined by a set of rules [8].

A tree has a *root node* (also called “top of the tree” or “first node”) whose *descendant nodes* (known as “daughters”) can be divided into *terminal* and *split nodes*. Leaf nodes, representing the last level of nodes, contain the final classification. Intermediate nodes are called “hidden” layers.

Input data consist of a response variable *Y* (e.g., an indicator variables for patients) and a set of explanatory variables 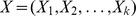 with fixed dimensionality *k*, where *X_i_* can be continuous, categorical ordinal/non ordinal, possibly including missing values.

A decision tree is generated according to the following algorithm [8]. At each node, you should.

1. examine every allowable split on each predictor variable (binary splits are generated by binary questions);
2. select and execute the “best” of the splits;
3. stop splitting on a node when some stopping rule is satisfied.

Binary trees, also chosen in our analysis, are the most popular type of tree. In a binary tree, by convention if the answer to a question is “yes”, the left branch is selected. The same question may appear more than one time in the network (see Figure 4).

To summarize, at each node, the tree algorithm searches through the variables one by one, beginning with *X_1_* and continuing up to *X_k_*. For each variable it finds the best split then it compares the *k* best single variable splits and selects the best of these. Steps 1 and 2 are then applied again to each of the daughter nodes and so on thus arriving at the full tree.

In Step 2, to select the “best” split, criteria based on indexes of entropy are commonly applied.

With reference to Step 3, in every recursive algorithm a stopping criterion must be defined to get an informative good tree. In the case of decision trees, it is crucial to decide when to stop trying to split nodes.

If not stopped, the tree algorithm will extract all the information from the data, so that resulting tree will fit random error or noise instead of describing underlying relationships among variables. A standard solution to this problem is to stop generating new split nodes when subsequent splits only result in very little overall improvement of the prediction.

Usually, splitting stops when each child nodes would contain less than five data points, or when splitting increases the information by less than some threshold.

One of the main drawbacks of decision trees is overfitting. In many situations, tree tends to grow too big and have too few data points in each terminal node to make the study worthwhile. To overcome overfitting problem, trees are then recursively pruned. For details on different pruning methods, see Breiman et al. (1984) [8].

### CART Analysis

The CART methodology is known as *binary* (i.e., parent nodes are always split into exactly two child nodes) and *recursive* (i.e., the process can be repeated by treating each child node as a parent) partitioning [35]. The standard criterion used in CART to get the best split to differentiate observations based on the dependent variable is the Gini rule, i.e., a measure of how well the splitting rule separates the classes contained in the parent node.

Variables are not selected in advance: CART algorithm is able to recognize the most significant variables and eliminate the non significant ones.

Of course, as mentioned in the previous section, when constructing a tree, crucial steps include deciding how to grow the tree, how to stop growing, and how to prune the tree to increase generalization [8]. Once the tree building algorithm has stopped, it is always useful to further evaluate the quality of the prediction of the current tree in samples of observations that did not participate in the original computations, applying for example cross-validation and *V*-fold cross-validation approaches. These methods are used to “prune back” the tree, i.e., to select a simpler tree, equally accurate for predicting or classifying “new” observations. To summarize, there are three important aspects in the construction of a tree [8], [36].

1. Split selection rule: at each node, choose split maximizing decrease in impurity (e.g. Gini index, entropy, misclassification error).
2. Split-stopping rule: grow large tree, prune to obtain a sequence of subtrees, then use cross-validation to identify the subtree with lowest misclassification rate.
3. Class assignment rule: for each terminal node, choose the class with the majority vote.

CART analysis is a powerful nonparametric and robust technique with significant potential and clinical utility. It is intended to identify distinct population subgroups and cannot provide the estimation of net effects of a single independent variable [37]. For the latter purpose, logistic regression techniques have been widely used, in order to estimate the “average” effect of an independent variable on the probability of being in a certain group, given also a set of other factors. Hence, if the purpose of the analysis is to quantify the influence of covariates on the outcome, CART analysis is not an adequate tool and regression techniques should be preferred in this type of situation. A toy example is shown in Figure 6, B.

## Supporting Information

File S1 **Figure S1 and Table S1.** Figure S1, “plotcp” function allows to plot mean and standard deviation of the errors in the cross-validated prediction of the “rpart” object shown in Figure 5 of the main document. Table S1, The cptable contains the mean and standard deviation of the errors in the cross-validated prediction for fitted trees (see Figure 5 of the main document and Figure S1 in File S1).
(PDF)
