# Supplementary material for: A Nonparametric Procedure for Defining a New Humoral Immunologic Profile in a Pilot Study on HIV Infected Patients
Source: PLoS One. 2013 Mar 22;8(3):e58768. doi: 10.1371/journal.pone.0058768 (PMC3606389; doi:10.1371/journal.pone.0058768)
Supplement: File S1 — Figure S1 and Table S1. Figure S1, “plotcp” function allows to plot mean and standard deviation of the errors in the cross-validated prediction of the “rpart” object shown in Figure 5 of the main document. Table S1, The cptable contains the mean and standard deviation of the errors in the cross-validated prediction for fitted trees (see Figure 5 of the main document and Figure S1 in File S1). (PDF) [file pone.0058768.s001.pdf]

Figure S1

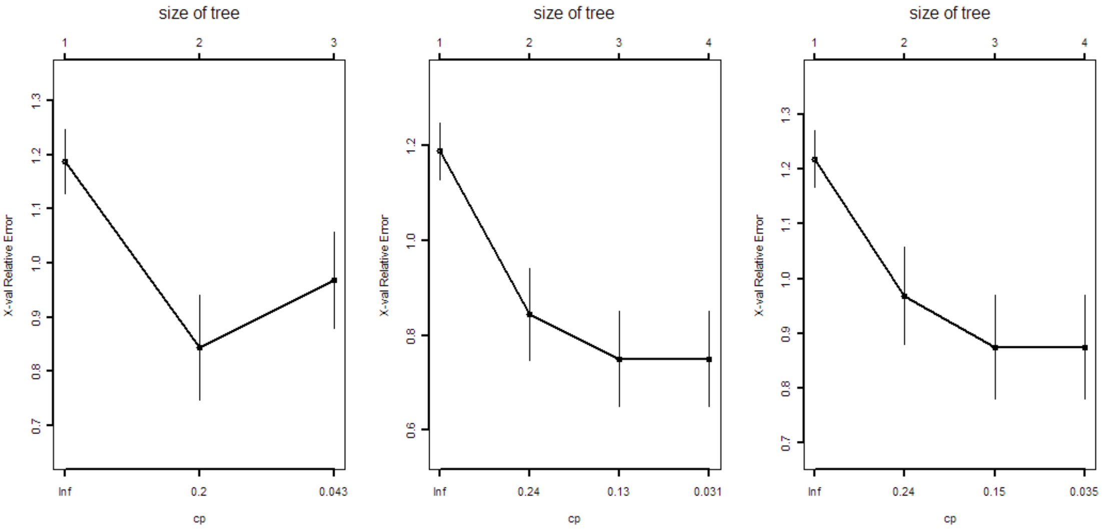

Table S1

Tree 1	CP	nsplit	rel error	xerror	xstd
1	0.21875	0	1.00000	1.18750	0.059449
2	0.18750	1	0.78125	0.84375	0.097040
3	0.01000	2	0.59375	0.96875	0.089044
Tree 2	CP	nsplit	rel error	xerror	xstd
1	0.31250	0	1.00000	1.18750	0.059449
2	0.18750	1	0.68750	0.84375	0.097040
3	0.09375	2	0.50000	0.75000	0.100223
4	0.01000	3	0.40625	0.75000	0.100223
Tree 3	CP	nsplit	rel error	xerror	xstd
1	0.3125	0	1.0000	1.21875	0.052158
2	0.1875	1	0.6875	0.96875	0.089044
3	0.1250	2	0.5000	0.87500	0.095470
4	0.0100	3	0.3750	0.87500	0.095470
